# Precision in Immune Management: Balancing Steroid Exposure, Rejection Risk, and Infectious Outcomes in Adult Kidney Transplant Recipients

**DOI:** 10.3390/jpm14111106

**Published:** 2024-11-14

**Authors:** Avery N. Koi, John C. Johnson, Trine L. Engebretsen, Muhammad A. Mujtaba, Alfred Scott Lea, Heather L. Stevenson, Michael L. Kueht

**Affiliations:** 1 John Sealy School of Medicine, The University of Texas Medical Branch, Galveston, TX 77555-0609, USA; 2 Department of Surgery, Division of Multiorgan Transplant and Hepatobiliary Surgery, The University of Texas Medical Branch, Galveston, TX 77555-0609, USA; 3 Department of Medicine, Division of Transplant Nephrology, The University of Texas Medical Branch, Galveston, TX 77555-0609, USA; 4 Department of Medicine, Division of Infectious Disease, The University of Texas Medical Branch, Galveston, TX 77555-0609, USA; 5 Department of Pathology, Division of Transplant Pathology, The University of Texas Medical Branch, Galveston, TX 77555-0609, USA

**Keywords:** kidney transplantation, immunosuppression, corticosteroids

## Abstract

Background/Objectives: With kidney transplant immunosuppression, physicians must balance preventing rejection with minimizing infection and malignancy risks. Steroids have been a mainstay of these immunosuppression regimens since the early days of kidney transplantation, yet their risks remain debated. Our study looks at the clinical outcomes of patients undergoing early steroid withdrawal (ESW) vs. steroid continuous (SCI) maintenance immunosuppression in adult kidney transplant recipients. Methods: A retrospective case-control study, utilizing propensity score-matching, was performed using the US Collaborative Network Database within TriNetX to evaluate renal transplant outcomes at one year in first-time kidney transplant adult patients (>18 years old) who were prescribed an ESW regimen (no steroids after post-transplant day 7 with maintenance tacrolimus [tac] + mycophenolic acid [MMP]/mycophenolate mofetil [MMF]) vs. SCI (tac + MMF/MMP + prednisone). Cohorts were matched on demographics, comorbidities, previously described risk factors for rejection, and induction immunosuppression. Primary outcomes included viral infections, pyelonephritis, and sepsis. Secondary outcomes included renal transplant rejection, death-censored allograft failure (eGFR < 15 mL/min), patient mortality, delayed graft function, and diabetes mellitus. Results: A total of 2056 patients were in each cohort after matching (mean age: 50.7–51 years, 17.9–20.0% African American, 60–60.6% male.) The SCI cohort had a significantly higher cumulative incidence of composite viremia (18 vs. 28.1%, ESW vs. SCI, *p* < 0.01) driven by CMV, EBV, and BK virus. Post-transplant diabetes mellitus was significantly higher in the SCI cohort (3.21% vs. 5.49%, ESW vs. SCI, *p* < 0.01). Delayed graft function was also higher in the SCI cohort (19.55% vs. 22.79%, ESW vs. SCI, *p* < 0.01). Pyelonephritis (2.3 vs. 4.91%, ESW vs. SCI, *p* < 0.01) and sepsis (2.15 vs. 5.95%, ESW vs. SCI, *p* < 0.01) were higher in the SCI cohort. Rejection rates were similar between ESW and SCI (29 vs. 31%, ESW vs. SCI, *p* = 0.41). There were significantly higher incidences of graft failure (4.9 vs. 9.9%, ESW vs. SCI, *p* < 0.01) and mortality (0.8 vs. 2.1%, ESW vs. SCI, *p* < 0.01) in the SCI cohort. Conclusions: This well-matched case-control study suggests that ESW is associated with lower infectious outcomes, mortality, and graft failure without increasing rejection risk, supporting the potential benefits of ESW in kidney transplant patients.

## 1. Introduction

Kidney transplantation remains the optimal, curative long-term treatment for end-stage kidney disease (ESKD). Nonetheless, there are far more people on the transplant waiting list than organs available. In 2023, ~27 thousand kidney transplant surgeries were conducted in the United States, while nearly 90 thousand people were still on the waiting list [1]. With the immunosuppression that accompanies transplantation, physicians must strike an intricate balance between preventing allograft rejection and minimizing the risks of infection or malignancy. 

Steroids have been a mainstay of transplant immunosuppression since the early days of kidney transplantation, yet the debate over their risks versus benefits has persisted as long. While steroids have an established role in preventing acute rejection, long-term steroids have a vast side effect profile that includes an increased risk of infection, decreased bone density, avascular necrosis, delayed wound healing, and post-transplant diabetes mellitus [2]. Because of this, many people have questioned the necessity of steroids post-transplant, especially with the widespread use of newer immunosuppression agents such as tacrolimus and mycophenolate. Most studies have indicated no significant difference in long-term outcomes, such as mortality, graft loss, and graft failure, among patients undergoing early steroid withdrawal, even in the studies where ESW was linked to a higher incidence of acute rejection [2,3]. Additional studies dating back to the 1980s show that early steroid withdrawal and/or avoidance in kidney transplant recipients does not adversely impact patient outcomes and decreases infectious outcomes [4,5,6]. The most recent and comprehensive study on the safety and efficacy of early steroid withdrawal in kidney transplant recipients is the Harmony trial, which showed the efficacy and beneficial safety outcomes, especially in reducing diabetes mellitus, of employing ESW in low immunologic risk patients at 1 and 5-year follow-ups [7,8]. Despite these data, many physicians still remain cautious and use continuous steroids as part of their treatment regimen for kidney transplant recipients. This exemplifies a need for more large-scale research and investigation into the risks and benefits of steroid use in this population, as well as physicians’ views surrounding ESW, to further shape clinical practices. 

Another key factor in the immunosuppression regimen for kidney transplant recipients is induction therapy, which is high-dose immunosuppressant agents that are administered typically before/during the transplant procedure and/or in the following days. In patients undergoing early steroid withdrawal (ESW), induction immunosuppression plays a particularly critical role in mitigating the risk of acute rejection. Interleukin-2 receptor antagonists, such as basiliximab, are typically the first-line agents for induction immunosuppression in patients at low immunologic risk. For those at high immunologic risk, lymphocyte-depleting agents, primarily antithymocyte globulin (ATG) and alemtuzumab, are commonly used. Immunologic risk must be carefully considered when choosing an induction immunosuppression agent for patients prescribed an ESW regimen, as it plays an even bigger role in preventing acute rejection.

In summary, numerous studies demonstrate that ESW offers benefits and comparable outcomes to SCI in kidney transplant recipients. Induction therapy is especially important in this population for reducing the risk of acute rejection. Current publications on early steroid withdrawal include randomized controlled trials (RCTs) and retrospective case controls with small sample sizes. Our study expands on the current knowledge and has a larger sample size of patients across the United States. This study also has the advantage compared to previous studies of using real-world patient data from electronic medical records across multiple healthcare organizations and the ability to propensity-score match patients on clinical characteristics that increase the risk for rejection. This study specifically explores the effect of early steroid withdrawal (ESW) versus steroid continuous immunosuppression (SCI) in adult patients (>18 years old) on the incidence of viral infections, pyelonephritis, sepsis, rejection, mortality, death-censored graft failure, delayed graft function (DGF), and post-transplant diabetes mellitus. 

## 2. Materials and Methods

### 2.1. Population Data

Our study analyzed the TriNetX “US Collaborative Network” to select first-time adult renal transplant recipients who underwent the procedure any time on or before 31 December 2020, which contains ~113 million individual electronic medical record (EMR) datasets across 64 healthcare organizations (HCOs) within the United States [9]. The database contains de-identified patient data and limits the information users can obtain to basic demographics, diagnostic, procedural, laboratory, genomic, oncological, and medication codes associated with individual EMRs, as well as limited information regarding geographic data. A thorough description of the platform and its validation is described by Palchuk et al. [10]. The TriNetX platform has been validated, and the results have shown its ability to replicate randomized controlled trial results using its real-world patient data [11,12]. TriNetX complies with the Health Insurance Portability and Accountability Act (“HIPPA”), the General Data Protection Regulation, the EU Data Protection Law, and Regulation 2016/679, such that patient identifiers and associated protected health information are not disclosed to its users. Also, to access the database, users must complete a Data Use Agreement and Collaborative Institutional Training Initiative (CITI Program) training at our institution. IRB approval was not required for this study due to the above reasons. Coding systems utilized for this analysis included Current Procedural Terminology (CPT) codes, International Classification of Diseases, Tenth Revision, Clinical Modification (ICD-10-CM) codes, ICD-10 Procedure Coding System (ICD-10-PCS) codes, RxNorm Technical Documentation (RxNorm) codes, and Logical Observation Identifier Names and Codes (LOINC) codes. 

### 2.2. Cohort Design 

Our study selected renal transplant recipients who were ≥18 years old at the time of undergoing transplantation between 31 December 2000 and 31 December 2020. Patients were excluded from the analysis if they had received a prior kidney transplant, another solid organ transplant (liver, pancreas, intestine, heart, lung), or a hematologic stem cell transplant anytime up to 1 day before the renal transplant procedure. Additionally, recipients who had undergone another solid organ transplant or hematologic stem cell transplant procedure on the same day as the renal transplant procedure or up to one year after were excluded from this analysis, ensuring that the cohorts included only first-time kidney transplant recipients. Additionally, patients were excluded for any history of diagnosed viral hepatitis or human immunodeficiency virus (HIV) infection at any time before the renal transplant procedure. In summary, our initial cohort narrowed the database network to adult, viral hepatitis-/HIV-, first-time renal transplant-only recipients. 

**Figure 1:**
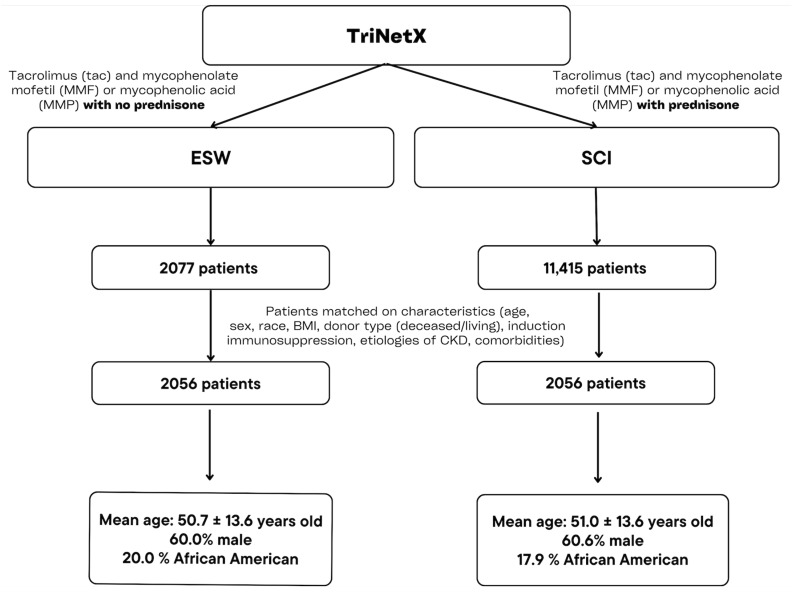
Methods.

From there, we narrowed the cohort further by selecting recipients who had been placed on tacrolimus (tac) AND mycophenolate mofetil (MMF) or mycophenolic acid (MPA) maintenance immunosuppression within the first post-transplant year. Recipients must have had ≥4 instances of tac + MMF/MPA in the EMR within this first transplant period. Recipients were excluded if they had any associated RxNorm code for alternate maintenance immunosuppression agents, including belatacept, azathioprine, cyclosporine, sirolimus, and everolimus within the first post-transplant year. We defined early steroid withdrawal (ESW) maintenance immunosuppression as no prednisone administration within the EMR at any time after 7 days post-renal transplant. Steroid continuous immunosuppression (SCI) was defined as ≥4 instances of prednisone within the EMR from 7–365 days post-renal transplant. This process identified 2077 ESW adult renal transplant recipients and 11,415 SCI adult renal transplant recipients (Figure 1). All associated codes used for defining ESW and SCI cohort criteria can be found in Supplementary S1.

### 2.3. Propensity Score Matching Variables 

Twenty-four recipient characteristics (codes) up to 10 years prior to the renal transplant procedure were matched in this study design. Codes with ≥50 recipients (in either cohort) were included in this analysis. Recipients were matched on the following demographic characteristics: age at the time of the renal transplant procedure (age at index); documented specified male sex; and documented race (Black or African American). Recipients were matched on previously described risk factors for rejection and coronary artery disease, including focal segmental glomerulosclerosis (FSGS), diffuse mesangial proliferative glomerulonephritis, systemic lupus erythematosus (SLE), diabetes mellitus status, and overweight and obesity status [13,14,15,16]. We further matched recipients on the type of induction immunotherapy: thymoglobulin; basiliximab; or alemtuzumab. Recipients were matched on the type of graft received: deceased; living; or unspecified (deceased or living). For laboratory codes, we factored in the recipient-calculated panel reactive antibodies (cPRA) in serum and HLA Class I Antibody, Panel-reactive Antibody (PRA) (86830 by LOINC), as well as recipient ABO and Rh [type] presence, cytomegalovirus (CMV) IgG antibody presence, Epstein–Barr virus (EBV) IgG antibody presence, and varicella–zoster virus (VZV) antibody presence. Prior exposure to immunosuppressive medications, rituximab, cyclophosphamide, and hydroxychloroquine were factored into the matching analysis. TriNetX offers limited information regarding donor characteristics. Supplementary S1 outlines codes used for the propensity-score matching analysis. 

### 2.4. Primary Analysis: Viral Infectious Outcomes and Graft Outcomes

The primary analysis aimed to explore infectious-related and graft-related outcomes from 14 to 365 days post-renal transplant procedure (1st post-transplant year). Outcomes at one-year follow-up were chosen, as this is the time patients are at the highest risk of opportunistic infections, which are the primary outcomes of this study. Virologic outcomes aimed to observe one-year incidences (14–365 days post-renal transplant) of diagnosed (by ICD-10-CM) or viremic episodes (≥1000 units/mL or ≥3 log copies/mL by LOINC) within the first post-transplant year. The primary virologic outcomes were CMV, EBV, JC, BK, and VZV virus. Virologic outcomes were composited using all codes (Supplementary S1) into a “Composite Viremia” outcome. We also looked at kidney infection outcomes, which included diagnosis by ICD-10-CM of “kidney transplant infection” or “acute pyelonephritis”. Sepsis was identified by ICD-10-CM code “sepsis, unspecified organism”. 

We defined allograft rejection as a composite of diagnosed rejection and/or treatment for rejection (thymoglobulin, methylprednisolone (solumedrol) ≥ 100 mg, or IVIg). TriNetX does not provide the ability to determine if the rejection episode is biopsy-proven. Analysis of primary outcomes began at day 14 post-transplant to account for thymoglobulin being both a method of induction immunosuppression and an outcome of interest. We further explored incidences of death-censored allograft failure within the 1st post-transplant year. Allograft failure was defined as an estimated glomerular filtration rate (eGFR) by creatinine-based formula (CKD-EPI) ≤ 15 mL/min/1.73 m^2^. Lastly, recipient mortality was defined as “deceased” according to TriNetX coding systems (Supplementary S1). 

Incidences of delayed graft function (DGF) were also assessed. DGF was defined using the widely accepted definition of DGF: any instance of hemodialysis within 1 week (day 1–day 7) following the kidney transplantation [17,18]. 

Incidences of diabetes mellitus were also included in this analysis. Diabetes mellitus was defined as a hemoglobin A1C value > 6.5%.

### 2.5. Descriptive Outcomes Analysis 

Descriptive outcomes analyzed renal function and tacrolimus/mycophenolate exposure within the first post-transplant year. For measures of renal function, recipient eGFR was measured at 1 month, 3 months, 6 months, and 12 months post-renal transplant, and the most recent eGFR within the first post-transplant year EMR was factored into a 14–365-day analysis. Similar time points were used for assessing serum tacrolimus blood levels (by LOINC codes) within the first post-transplant year. The aim of these descriptive outcomes was to assess any underlying differences in tacrolimus immunosuppression and eGFR within the first post-transplant year. All lab measurements were the most recent lab values unless otherwise stated (Supplementary S1). 

### 2.6. Statistical Analysis 

Propensity score matching (1:1) with greedy nearest neighbor matching was used to create balanced cohorts. Propensity-score matching variables with a *p*-value of ≥0.05 were considered well-matched. Incidences of rejection, graft failure, mortality CMV viremia, EBV viremia, BK viremia, JC viremia, VZV viremia, composite viremia, and DGF were calculated using Kaplan–Meier (KM) analysis. KM analysis provided Log-Rank Test analyses and Hazard Ratio and proportionality analyses with a *p*-value ≤ 0.05 considered statistically significant. For descriptive laboratory outcomes (renal eGFR measures and serum tacrolimus levels) that were continuous values, *t*-test analysis was performed with a *p*-value ≤ 0.05 being considered statistically significant. 

## 3. Results

### 3.1. Propensity Score Matching Results

After matching, there were 2056 recipients in each cohort (Table 1). The age at transplant difference was normalized, and the cohorts were well-matched on sex and race. The mean follow-up was 359.41 ± 38.27 days in the ESW cohort and 360.89 ± 29.46 days in the SCI cohort. There were no significant differences between the cohorts for pre-transplant comorbid conditions (*p* > 0.05). While there was an imbalance of living and deceased donors (24% LD in ESW and 14% LD in SCI) prior to matching, after matching, the absolute difference remained small (23.6% LD in ESW and 20.7% LD in SCI) with a significant *p*-value of 0.02. While statistically significant, there is only a 2.9% absolute difference in LDs, and deceased donor percentages were equivocal between the cohorts (22.1% LD in ESW and 22.5% LD in SCI). The cohorts were well-matched on induction immunosuppression, pre-transplant immunosuppressive exposure, and mean cPRA (*p* > 0.05). HLA-A+B+C (class I) Ab in serum were marginally significantly higher in the ESW cohort after matching (2.8% vs. 2.4%, *p* = 0.041.) Table 1 gives a full breakdown of pre- and post-matching characteristics observed between the ESW and SCI cohorts. 

Any propensity score matching result with *n* = 10 was excluded from the table because TriNetX queries did not return a value lower than 10 to protect patient data and de-identification. After matching, results with less than 10 samples included Nephrotic syndrome with focal and segmental glomerular lesions, cyclophosphamide, and rituximab.

Table 1 Recipient population characteristics up to 10 years prior to the renal transplant procedure in the ESW and SCI cohorts before and after propensity score-matching.

### 3.2. Infectious Outcomes

Infectious outcomes are displayed in Table 2.

### 3.3. Graft and Recipient Outcomes 

Kaplan–Meier analysis showed that ESW did not have a significantly different cumulative incidence of rejection compared to SCI (28.99% vs. 30.97%, *p* = 0.41). No significant difference was observed in the hazard of rejection between the cohorts. There was a significantly higher incidence of graft failure (9.9% vs. 4.92%, *p* < 0.01) and mortality (2.11% vs. 0.83%, *p* < 0.01) in the SCI cohort. The ESW cohort had an approximately 50% (39–62%) hazard reduction for death-censored graft failure and approximately 60% (31–78%) hazard reduction for mortality compared to SCI during the first post-transplant year. Delayed graft function, defined as hemodialysis 1–7 days post-transplant, was higher in the SCI cohort (5.49% vs. 3.21%, *p* < 0.01). Additionally, diabetes mellitus was significantly higher in the SCI cohort (22.79% vs. 19.55%, <0.01) compared to the ESW cohort. The graft and recipient outcomes are depicted in Table 3.

**Figure 2:**
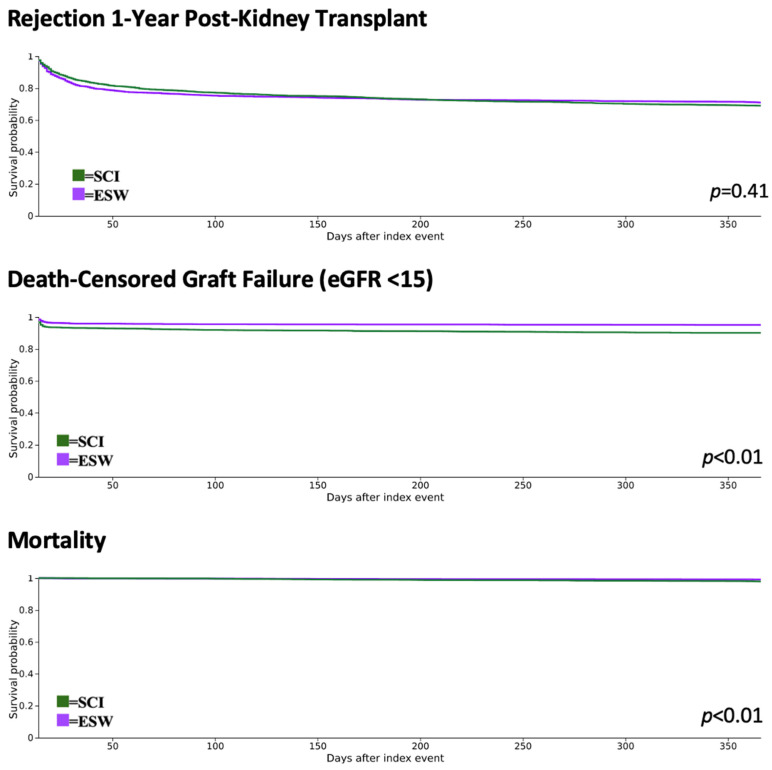
Kaplan–Meier event-free survival curves for rejection, death-censored graft failure (eGFR < 15), and mortality one year post-kidney transplant.

The Kaplan–Meier event-free survival curves depicted in Figure 2 show that the probability of remaining rejection-free at the end of one-year did not significantly differ between the two cohorts (71.01% in ESW vs. 69.03% in SCI, *p* = 0.41), while the probability of remaining mortality-free was higher in the ESW cohort (99.17% in the ESW vs. 97.89% in the SCI cohort, *p* < 0.01), and the probability of remaining free of graft failure was higher in the ESW cohort (95.08% in ESW vs. 90.10% in SCI, *p* < 0.01.)

### 3.4. Descriptive Outcomes 

**Figure 3:**
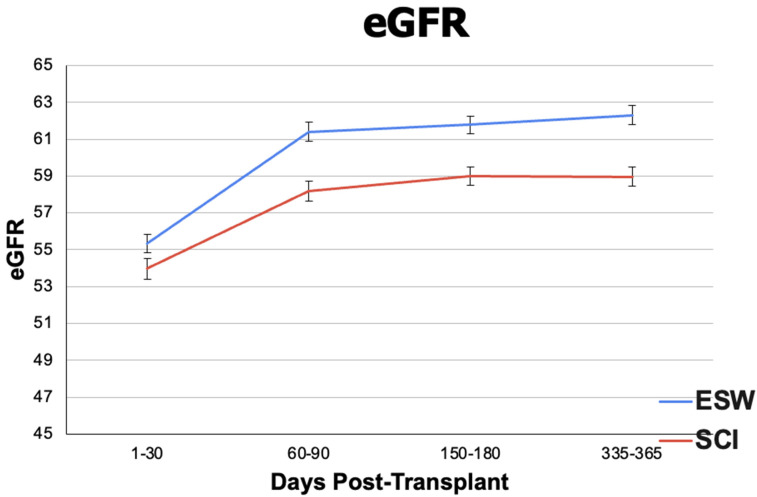
Mean eGFR (mL/min/1.73 m^2^) ± standard error at 1 month (1–30 days), 3 months (60–90 days), 6 months (150–180 days), and 12 months (335–365 days) post-renal transplant procedure between the ESW cohort (blue line) and SCI cohort (red line).

**Figure 4:**
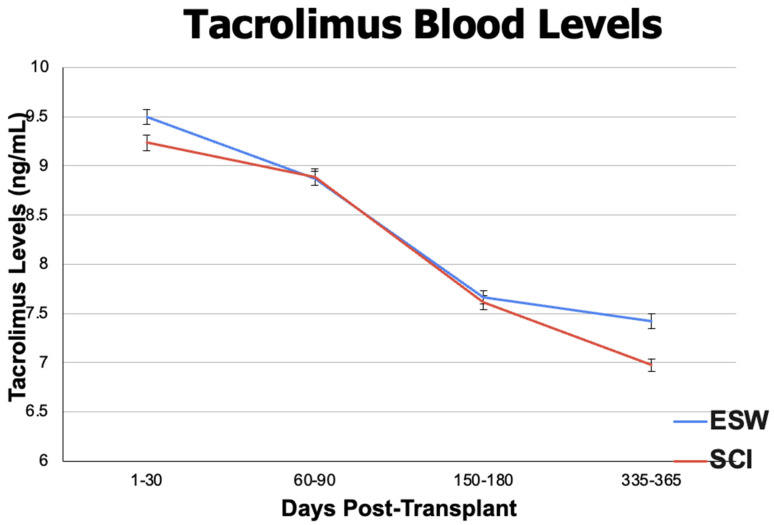
Mean tacrolimus (ng/mL) blood levels ± standard error at 1 month (1–30 days), 3 months (60–90 days), 6 months (150–180 days), and 12 months (335–365 days) post-renal transplant procedure between the ESW cohort (blue line) and SCI cohort (red line).

**Figure 5:**
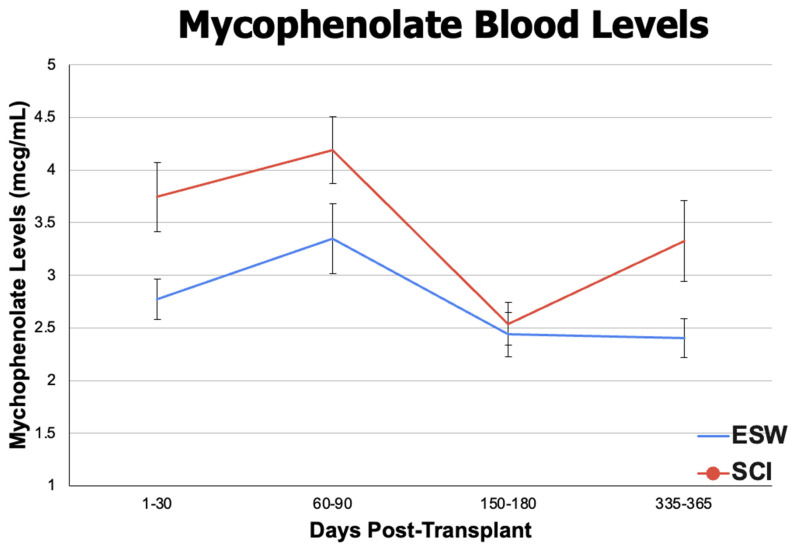
Mean mycophenolate (mcg/mL) blood levels ± standard error at 1 month (1–30 days), 3 months (60–90 days), 6 months (150–180 days), and 12 months (335–365 days) post-renal transplant procedure between the ESW cohort (blue line) and SCI cohort (red line).

Descriptive outcomes included eGFR to evaluate kidney function and tacrolimus + mycophenolate blood levels to see if there was a difference in immunosuppression levels between the cohorts. When analyzing eGFR (mL/min/1.73 m^2^), the ESW cohort had significantly higher mean eGFR levels at 3 months (61.389 ± 18.827 in ESW, 58.179 ± 21.214 in SCI, *p* < 0.01), 6 months (61.783 ± 18.397 in ESW, 58.994 ± 20.162 in SCI, *p* < 0.01), and 12 months (62.308 ± 18.706 in ESW, 58.958 ± 19.889 in SCI, *p* < 0.01) post-renal transplant, although the SCI cohort remained in normal post-transplant physiologic range throughout this time (Figure 3). For mean tacrolimus serum levels (ng/mL), the ESW cohort had a significantly higher mean serum level within the first post-transplant month (9.500 ± 3.062 in ESW, 9.236 ± 3.313 in SCI, *p* = 0.02) and at 12 months post-transplant (7.420 ± 2.815 in ESW, 6.977 ± 2.466 in SCI, *p* < 0.01). No difference was observed at 3 months and 6 months (Figure 4). Mycophenolate levels were significantly higher in the SCI cohort at 1-month post-transplant (2.775 ± 1.771 in ESW, 3.745 ± 3.529 in SCI, *p* < 0.01), but no difference was observed at 3, 6, or 12 months post-transplant (Figure 5). Results of eGFR, tacrolimus levels, and mycophenolate levels at different time intervals are depicted in Figure 3 (eGFR) and Figure 4 (tacrolimus level) and Figure 5 (mycophenolate levels) to illustrate changes over time.

## 4. Discussion

This well-matched retrospective multicenter case-control study of one-year clinical outcomes of first-time kidney transplant recipients undergoing early steroid withdrawal or steroid continuous maintenance immunosuppression found that ESW was not associated with an increased incidence of rejection and had a lower incidence of mortality and graft failure at 1 year post-transplant. Additionally, the ESW cohort had lower incidences of composite viremia (CMV, EBV, BK, JC, VZV), pyelonephritis, and sepsis. 

Without protecting against rejection or graft loss, infectious outcomes were significantly higher in the steroid continuous cohort. Steroids have been found to increase the incidence of infection in a dose-dependent manner and are one of the leading causes of death with a functioning graft along with cardiovascular disease [19]. In a study by Opelz and Döhler, patients with a steroid dose >0.10 mg/kg/day had a significantly higher risk of death with a functioning graft compared to patients on a lower dose, whereas this was not seen with the other immunosuppression agents cyclosporine, tacrolimus, azathioprine, or mycophenolic acid. 

This unique effect of corticosteroids was apparent in our study. While we do not have the exact results for death with a functioning graft, we looked at mortality and graft failure rates. The SCI cohort had significantly higher mortality and graft failure rates with similar allograft rejection rates. These data suggest that rejection may not be the main driver of graft loss, although much of our treatments are aimed at the prevention of rejection. While we cannot be entirely certain of the reason, there were significantly higher infectious outcomes in the SCI cohort, notably sepsis and pyelonephritis, which could be a major source of mortality and graft failure. Within the outcome of pyelonephritis, most patients also had a specific diagnosis of “kidney transplant infection”, which was higher in the SCI cohort, further supporting the hypothesis of infection causing graft failure. While more investigation is warranted, it is apparent that infections are a major source of mortality and graft failure associated with steroids. Many other studies support this finding and have found that lower steroid exposure decreases adverse outcomes, with infection being the most significant [19,20,21,22]. 

Induction immunosuppression with ATG is a known risk factor for CMV infection in kidney transplant recipients. Universal prophylaxis is the preferred management of patients on ATG as set forth by the international CMV consensus guidelines [23]. Our study accounted for this risk factor of CMV by matching patients on induction therapy type as well as CMV seropositivity, and the cohorts were well-balanced for these risk factors. The SCI cohort had a significantly higher incidence of CMV compared to ESW. Although TriNetX had limited information on the dosage of ATG patients received, it might be possible that the ESW cohort received higher dosages of ATG for induction therapy, but even so, CMV incidence was significantly lower in ESW than in the SCI cohort.

Our study found that the ESW cohort did not have an increased incidence of rejection compared to the SCI cohort at 1 year post-transplant. Research on the necessity of steroid use in kidney transplant recipients has been ongoing since the 1980s, with numerous studies showing that steroids may be safely withdrawn early in the post-transplant period to avoid adverse effects [4,5,6]. Randomized controlled trials (RCTs) with smaller sample sizes but longer follow-up (10–15 years) have shown that rapid steroid withdrawal did not negatively impact patient survival; in contrast, our study found that there was actually a higher patient survival rate in the ESW cohort vs. SCI cohort [2]. These RCTs also found that rapid steroid withdrawal was associated with higher graft survival rates in patients who received a deceased donor kidney, while there was no difference between patients who received a living donor kidney [21,22]. Similarly, our results showed increased death-censored graft survival in the ESW cohort compared to the SCI cohort, though patients were matched on donor type (living/deceased/unknown) and not evaluated separately in the results. Other existing sources of the literature on early steroid withdrawal in kidney transplant recipients have shown that it might be associated with an increased incidence of biopsy-proven acute rejection, but the long-term patient outcomes are not inferior, and patients benefit from fewer adverse effects [2,24,25]. Our study showed similar rejection rates between the two groups at one year post-renal transplant. Given these findings, it would appear safe for average immunologic risk patients to be withdrawn from steroids early to avoid the drug’s adverse outcomes, with infection being a major concern. 

Diabetes mellitus is another major adverse effect of long-term use of steroids, leading to significant morbidity and increasing the risk of cardiovascular disease. Our study found that ESW was associated with a lower incidence of diabetes mellitus compared to the SCI cohort. This was also shown in the Harmony trial, which showed the safety and efficacy of ESW at 1 and 5-year follow-ups, with the major benefit of reduced incidence of diabetes mellitus [7,8].

The incidence of delayed graft function (DGF) was significantly higher in the SCI compared to the ESW cohort. DGF has been associated with worse long-term graft function and survival, though some studies have conflicting results [26,27,28]. Nevertheless, DGF could have played a role in the inferior outcomes of mortality and graft failure in the SCI cohort.

In a study by Bae S. et al., ESW in patients with DGF was linked to worse outcomes, including higher rates of graft failure and rejection, while mortality was comparable to SCI [29]. In patients with immediate graft function, ESW was associated with increased rejection and graft failure rates but showed the potential benefits of lower mortality rates. This study, which used the elegant SRTR database, faced a major limitation of being unable to enact a strict definition of ESW. Patients were assigned to ESW if maintenance steroids were discontinued at hospital discharge, while all other patients were assigned to the steroid continuous cohort. Our study has the advantage of applying a strict definition of ESW as no steroids, defined as <four instances, after 7 days post-transplant, which only allows for a short regimen of steroids for treating a rejection episode.

Our descriptive outcomes included eGFR and immunosuppression levels. There was no clinically significant difference in eGFR between the cohorts, suggesting similar kidney functions. The absolute max difference between eGFR in the cohorts was 3 mL/min/1.73 m^2^, which clinicians would interpret as equivocal kidney function. The mean tacrolimus serum levels (ng/mL) between the cohorts were significantly different in the first week (9.500 +/− 3.062 in ESW, 9.236 +/− 3.313 in SCI, *p* = 0.02) and at 12 months post-transplant (7.420 +/− 2.815 in ESW, 6.977 +/− 2.466 in SCI, *p* < 0.01). However, from the clinical standpoint, a mean level of 9.500 vs. 9.236 and 7.420 vs. 6.977 is not a clinically significant difference, and physicians would not adjust dosing to grow from one value to the other because the levels provide relatively equivocal immunosuppression. With this reasoning, tacrolimus blood levels were relatively similar between the cohorts, limiting the confounding variable of over-immunosuppression with tacrolimus in ESW. 

While mycophenolate levels are not widely used clinically and are most commonly prescribed as a calculated standard dose, new research is looking at whether the concentration-based dosing would be beneficial. A new subject of research relevant to our study is the interaction between corticosteroids and mycophenolate. There seem to be conflicting results on whether glucocorticoids decrease mycophenolate mofetil bioavailability or if corticosteroids increase mycophenolic acid levels in the blood [30,31,32]. A study by Nourbakhsh et al. found that corticosteroids increased mycophenolic acid exposure in renal transplant patients on a conventional prednisolone + tacrolimus + mycophenolate mofetil regimen compared to a steroid-free regimen [31] In our study, mycophenolate levels were significantly higher at 1 month post-transplant in the SCI cohort and remained higher at 3, 6, and 12 months post-transplant, though it was not statistically significant. If corticosteroids indeed increase mycophenolic acid exposure, this can be an additional mechanism by which steroids increase infection in patients treated with the traditional steroid-based regimen (tac + MMF + corticosteroids.) This is a link that will need to be studied further, as the research is sparse.

Prior to matching, there was a large difference in cohort sizes between ESW (2077) and SCI (11,415). This reflects steroids being a mainstay in kidney transplant immunosuppression regimens and reinforces our point of this study to show that physicians remain hesitant to employ ESW despite numerous previous studies we have described above that show its safety and efficacy in low-immunologic risk patients. The patients in our cohorts also could have received a kidney transplant anytime between 31 December 2000 and 2020, with steroids being extremely common in the early 2000s. The future direction of this study would be to conduct a large-scale, multicenter randomized controlled trial in the United States and survey reasons why physicians remain hesitant to conduct ESW in low immunologic risk patients if the benefits have been shown and its safety established. It seems to be largely based on the increased incidence of acute rejection in patients on ESW, but studies have shown that this did not affect long-term patient outcomes, and patients benefited from fewer adverse effects [2,21,22].

There are many strengths to this study. Utilization of real-world patient data allowed us to employ clinical information for propensity-score matching across a broad range of patient characteristics, thereby minimizing confounding variables. Additionally, this study’s sample size was sufficiently large and included patient data from 64 HCOs across the U.S. to enable us to draw reliable and unbiased conclusions. 

Our study does not come without limitations. This study uses the TriNetX database, which, while excellent at obtaining data from a large sample population, is limited by reporting in the patient medical record. Rejection was one of the outcomes for this study, and TriNetX did not indicate whether the rejection was biopsy-proven or its severity. For this study, we used an ICD code of kidney transplant rejection as well as treatment for rejection with thymoglobulin or methylprednisolone (solumedrol) ≥100 mg, which could inadvertently include treatment for other severe cases of autoimmune diseases such as SLE, vasculitis, etc. Additionally, we were limited in the amount of donor-derived information we could obtain (donor age, donor cause of death, cold and warm ischemic times, donor serologies) from the database, although we tried to be as scrupulous as we could with the amount of recipient information available to us. Because many donor factors were not available for this study, we included infection as the primary outcome, as this was influenced, to a lesser extent, by donor factors than by recipient factors and the post-transplant course. Additionally, a major consequence of using steroids in the long-term maintenance immunosuppression regimen is infectious outcomes, which is a major source of morbidity and mortality. Steroid dosages in the SCI cohort were not factored into our analysis due to limited data. While TriNetX offered detailed information in regard to specific dosage parameters, nearly 45% of the SCI cohort had an “unspecified” dose given to them during this time, which limited the degree to which we could factor this in. Additionally, TriNetX only reports one lab value (LOINC 23905-3) for mycophenolate levels, which was used as one of the descriptive outcomes to approximate mycophenolate exposure. While AUC is the more accurate way to measure mycophenolate exposure, we were limited to using a single lab value as this is how the TriNetX database reports this data. Our study was retrospective in design and relied on accurate documentation that was out of our control.

Another limitation of using a database was in obtaining induction immunosuppressants used. Induction immunosuppression was factored into propensity-score matching, and there were equal proportions of patients receiving anti-thymocyte globulin, basiliximab, and alemtuzumab in each cohort. Unfortunately, not all patients had induction immunosuppressants recorded in the database, which was a limitation in using large databases, but the cohorts were well-matched on induction immunosuppression agents documented.

Due to this being a retrospective study using a national database, patients were not randomly selected into the two cohorts, and we had no jurisdiction in their assignments. Clinicians used their own clinical judgment to prescribe patients to either an ESW or SCI regimen. Additionally, the sample size for our study was determined by the data available in the TriNetX database rather than priori assumptions or calculations set by us. As this is a retrospective analysis utilizing an existing multicenter database, we leveraged the largest possible sample size that met our inclusion and exclusion criteria to maximize this study’s power and generalizability. We attempted to minimize the influence of selection bias in the ESW and SCI cohorts by incorporating an extensive list of risk factors into the matching algorithm. We ensured equal proportions of high and low immunologic risk recipients in each group by matching on immunologic ESRD etiologies, induction agents, desensitization procedures, cPRA, and age. Clinical situations that required desensitization were attempted to be captured by accounting for plasmapheresis, a procedure used in most immunologically risky scenarios, including ABOi and highly sensitized recipients. Nonetheless, this approach may leave room for unaccounted-for confounders, which is a limitation of this study. While our cohorts included both high and low-immunologic-risk patients, most studies on ESW have not looked at high-immunologic-risk patients, so a future direction from this study would be to assess outcomes of ESW in this population.

We additionally matched patients on HLA A+B+C (Class I) Antibody status, but TriNetX did not have information available on HLA DR mismatch. However, we extensively matched on other previously described risk factors for rejection and relied on clinicians’ expertise to take HLA DR mismatches into consideration when treating patients by employing appropriate interventions such as higher dose ATG induction therapy, which was part of propensity-score matching.

Another limitation of this study is that it was not possible to design the cohorts as “intention-to-treat”. As TriNetX is a real-world database not collected specifically for this study, it is challenging to ensure that the early steroid withdrawal (ESW) cohort remains strictly “intention to treat”. This is due to the inherent difficulty in understanding how frequently medication administrations, like steroids, were recorded by different physicians. TriNetX, however, offers tools such as the Treatment Pathways Analysis function, which helps to assess how long patients remained on specific medications post-transplant. For the original SCI cohort, we utilized this tool and found that the mean time on prednisone within the first post-transplant year was 350.724 ± 46.799 days, with a median of 363 days. The time to treatment was a mean of 11.113 +/− 35.731 days with a median of 3 days. This tool suggests that the majority of the SCI cohort started on prednisone within the first week post-transplant. While there were a few patients who began steroids later, this represents a very small subset of the cohort that is unlikely to significantly impact results. However, it is an important limitation to note, and the future direction for this study is to conduct a multicenter randomized controlled trial in centers across the United States as most current RTCs are single-center or outside the United States. This methodology does, however, have the advantage of making the ESW a pure cohort that has never received >4 doses of steroids past 7 days post-transplant. This makes the analysis of infectious outcomes, rejection rates, mortality, and graft failure in the ESW extremely accurate and reflective of real-life patients.

Our definition of rejection (previously published: Drugs Aging, 17 October 2024, PMID: 39417973) includes a composite of treatment for and diagnosis of rejection [32]. This aims to better capture the risks of rejection, which is not only graft injury but also the side effects of increased immunosuppression. Therefore, our reported rejection rates may be higher than reports that rely solely on biopsy-proven rejection.

Another limitation of this study is only differentiating race as “African American” and “others”, although other minorities also seem to suffer more from chronic kidney disease and face disparities that affect kidney transplant outcomes. Future directions from this study include an in-depth analysis of how race and ethnicity impact kidney transplant outcomes.

## 5. Conclusions

Based on this study, including continuous steroids in the post-kidney transplant maintenance immunosuppression regimen for first-time kidney transplant recipients was not associated with lower rates of rejection and was associated with more infectious complications, more graft loss, and more mortality. Early steroid withdrawal was associated with equal rejection rates but lower morbidity and mortality. Given these findings, adult kidney transplant recipients may benefit from lower steroid exposure, avoiding unintended consequences that increase graft failure and mortality without increasing rejection. 

## Figures and Tables

**Table 1:** Propensity Score Matching Results.

	Before Matching Mean ± SD; *n* (%)				After Matching Mean ± SD; *n* (%)			
	ESW *n* = 2077	SCI *n* = 11,415	*p*-Value	Std. Diff.	ESW *n* = 2056	SCI *n* = 2056	*p*-Value	Std. Diff.
Age at index	50.7 +/− 13.9	51.5 +/− 13.5	0.02	0.06	50.7 +/− 13.9	51.0 +/− 13.6	0.47	0.02
Black or African American	413 (20%)	3117 (27.3%)	<0.01	0.17	412 (20.0%)	367 (17.9%)	0.07	0.06
Male	1240 (60.0%)	6665 (58.5%)	0.18	0.03	1234 (60.0%)	1246 (60.6%)	0.70	0.01
Diagnosis								
Unspecified nephritic syndrome with focal and segmental glomerular lesions	44 (2.1%)	398 (3.5%)	<0.01	0.08	44 (2.1%)	37 (1.8%)	0.43	0.03
Chronic nephritic syndrome with diffuse mesangial proliferative glomerulonephritis	79 (3.8%)	490 (4.3%)	0.33	0.02	78 (3.8%)	71 (3.5%)	0.56	0.02
Systemic lupus erythematosus (SLE)	61 (3.0%)	501 (4.4%)	<0.01	0.08	61 (3.0%)	46 (2.2%)	0.14	0.05
Overweight and obesity	369 (17.9%)	2454 (21.5%)	<0.01	0.09	367 (17.9%)	357 (17.4%)	0.68	0.01
Diabetes mellitus	633 (30.7%)	4447 (39.0%)	<0.01	0.18	630 (30.6%)	644 (31.3%)	0.64	0.02
Procedure								
Backbench standard preparation of deceased donor renal allograft prior to transplantation	454 (22.0%)	3952 (34.7%)	<0.01	0.28	454 (22.1%)	463 (22.5%)	0.74	0.01
Backbench standard preparation of living donor renal allograft (open or laparoscopic) prior to transplantation	493 (23.9%)	1609 (14.1%)	<0.01	0.25	485 (23.6%)	425 (20.7%)	0.02	0.07
Backbench reconstruction of deceased or living donor renal allograft prior to transplantation	208 (10.1%)	1465 (12.9%)	<0.01	0.09	205 (10.0%)	170 (8.3%)	0.06	0.06
Labs								
Epstein Barr virus capsid IgG Ab [Presence] in Serum by Immunoassay	209 (10.1%)	1519 (13.3%)	0.03	0.196	206 (10.0%)	197 (9.6%)	0.075	0.176
Cytomegalovirus IgG Ab [Presence] in Serum or Plasma by Immunoassay	474 (23.0%)	1733 (15.2%)	<0.01	0.367	466 (22.7%)	417 (20.3%)	0.06	0.058
Varicella zoster virus IgG Ab [Presence] in Serum by Immunoassay	363 (17.6%)	971 (8.5%)	0.07	0.120	355 (17.3%)	307 (14.9%)	0.96	0.004
HLA-A+B+C (class I) Ab in Serum	60 (2.9%)	158 (1.4%)	0.003	0.379	58 (2.8%)	50 (2.4%)	0.041	0.412
Induction Immunosuppression								
Anti-thymocyte globulin	359 (17.4%)	4045 (35.5%)	<0.01	0.419	359 (17.5%)	316 (15.4%)	0.07	0.056
Basiliximab	160 (7.7%)	1230 (10.8%)	<0.01	0.105	160 (7.8%)	145 (7.1%)	0.372	0.028
Alemtuzumab	80 (3.9%)	206 (1.8%)	<0.01	0.125	77 (3.7%)	80 (3.9%)	0.807	0.008

**Table 2:** Infectious Outcomes. Cumulative incidences of CMV, BK, EBV, JC virus, VZV, composite viremia, pyelonephritis, and sepsis outcomes in ESW vs. SCI from 14 to 365 days post-renal transplant procedure.

Outcome	Cohort	Cumulative Incidence (%)	*p*	Hazard Ratio (95% Confidence Interval)
CMV	ESW	14.00%	<0.01	0.674 (0.580, 0.784)
	SCI	20.19%		
BK	ESW	4.22%	<0.01	0.508 (0.392, 0.660)
	SCI	8.14%		
EBV	ESW	0.39%	<0.01	0.227 (0.106, 0.490)
	SCI	1.72%		
JC Virus	ESW	0.00%	0.32	N/A
	SCI	0.05%		
VZV	ESW	0.64%	0.13	0.589 (0.297, 1.169)
	SCI	1.09%		
Composite Viremia (CMV, EBV, BK, JC, VZV)	ESW	17.97%	<0.01	0.607 (0.532, 0.692)
	SCI	28.09%		
Pyelonephritis	ESW	2.30%	<0.01	0.466 (0.330, 0.659)
	SCI	4.91%		
Sepsis	ESW	2.15%	<0.01	0.357 (0.255, 0.498)
	SCI	5.95%		

**Table 3:** Secondary outcomes. Cumulative incidences of rejection, graft failure, and mortality in ESW vs. SCI from 14 to 365 days post-renal transplant procedure.

	Cohort	Cumulative Incidence (%)	*p*	Hazard Ratio (95% Confidence Interval)
Rejection	ESW	28.99%	0.41	0.955 (0.854, 1.068)
	SCI	30.97%		
Death-censored Graft Failure	ESW	4.92%	<0.01	0.487 (0.383, 0.618)
	SCI	9.90%		
Mortality	ESW	0.83%	<0.01	0.394 (0.225, 0.691)
	SCI	2.11%		
Delayed Graft Function	ESW	3.21%	<0.01	0.577 (0.438, 0.76)
	SCI	5.49%		
Diabetes Mellitus	ESW	19.55%	<0.01	0.849 (0.752, 0.959)
	SCI	22.79%		

## Data Availability

The data presented in this study are available in TriNetX: https://live.trinetx.com/ accessed on 13 May 2024. This data is not openly available; users must request access to this database.

## Supplementary Materials

The following supporting information can be downloaded at https://www.mdpi.com/article/10.3390/jpm14111106/s1, Supplementary S1: Methods: Definition of Cohorts, 1:1 Propensity Score-Matching Criteria, and Outcomes Definitions.
