# Supplementary material for: Precision in Immune Management: Balancing Steroid Exposure, Rejection Risk, and Infectious Outcomes in Adult Kidney Transplant Recipients
Source: J Pers Med. 2024 Nov 14;14(11):1106. doi: 10.3390/jpm14111106 (PMC11595447; doi:10.3390/jpm14111106)
Supplement: Supplementary file 1 [file jpm-14-01106-s001.zip › jpm-3260497-supplementary.pdf]

Methods: Definition of Cohorts, 1:1 Propensity Score-Matching Criteria, and Outcomes Definitions

1) **Definition of Cohorts**

Adult Renal Transplant Recipients

Recipients must have been ≥ 18 years old at the time of their first renal transplant procedure and the record must have occurred in the EMR either on or before December 31, 2020, using the following codes:

1) CPT: 50360 - Renal allotransplantation, implantation of graft; without recipient nephrectomy (at least 18 years old at event)
2) CPT: 50365 - Renal allotransplantation, implantation of graft; with recipient nephrectomy (at least 18 years old at event)
3) ICD-10-PCS: 0TY00Z0 - Transplantation of Right Kidney, Allogeneic, Open Approach (at least 18 years old at event)
4) ICD-10-PCS: 0TY10Z0 - Transplantation of Left Kidney, Allogeneic, Open Approach (at least 18 years old at event)

Exclusion of prior renal transplant procedure/diagnosis or other organ transplant/diagnosis at least 1 day before the renal transplant procedure.

1) CPT:50360 - Renal allotransplantation, implantation of graft; without recipient nephrectomy
2) CPT:50365 - Renal allotransplantation, implantation of graft; with recipient nephrectomy
3) ICD10CM:Z94.0 - Kidney transplant status
4) CPT:32851 - Lung transplant, single; without cardiopulmonary bypass
5) CPT:32852 - Lung transplant, single; with cardiopulmonary bypass
6) CPT:32853 - Lung transplant, double (bilateral sequential or en bloc); without cardiopulmonary bypass
7) CPT:32854 - Lung transplant, double (bilateral sequential or en bloc); with cardiopulmonary bypass
8) CPT:33935 - Heart-lung transplant with recipient cardiectomy-pneumonectomy
9) CPT:38241 - Hematopoietic progenitor cell (HPC); autologous transplantation
10) CPT:38240 - Hematopoietic progenitor cell (HPC); allogeneic transplantation per donor
11) CPT:44135 - Intestinal allotransplantation; from cadaver donor
12) CPT:47135 - Liver allotransplantation, orthotopic, partial or whole, from cadaver or living donor, any age
13) CPT:48160 - Pancreatectomy, total or subtotal, with autologous transplantation of pancreas or pancreatic islet cells
14) CPT:48554 - Transplantation of pancreatic allograft
15) ICD10CM:Z94.4 - Liver transplant status
16) ICD10CM:Z94.7 - Corneal transplant status
17) ICD10CM:Z94.84 - Stem cells transplant status
18) ICD10CM:Z94.81 - Bone marrow transplant status
19) ICD10CM:Z94.5 - Skin transplant status
20) ICD10CM:Z94.1 - Heart transplant status
21) ICD10CM:Z94.2 - Lung transplant status

22) ICD10CM:Z94.83 - Pancreas transplant status
23) ICD10CM:Z94.6 - Bone transplant status
24) ICD10CM:Z94.82 - Intestine transplant status
25) ICD10CM:Z94.3 - Heart and lungs transplant status
26) ICD10CM:Z94.8 - Other transplanted organ and tissue status
27) ICD10CM:Z94.9 - Transplanted organ and tissue status, unspecified
28) ICD10PCS:0FY - Transplantation
29) ICD10PCS:02Y - Transplantation
30) ICD10PCS:0BY - Transplantation
31) ICD10PCS:0TY – Transplantation

Exclusion of any instance of other organ transplant/diagnosis at least 1 day after the renal transplant procedure using the following codes:

1) CPT:32851 - Lung transplant, single; without cardiopulmonary bypass
2) CPT:32852 - Lung transplant, single; with cardiopulmonary bypass
3) CPT:32853 - Lung transplant, double (bilateral sequential or en bloc); without cardiopulmonary bypass
4) CPT:32854 - Lung transplant, double (bilateral sequential or en bloc); with cardiopulmonary bypass
5) CPT:33935 - Heart-lung transplant with recipient cardiectomy-pneumonectomy
6) CPT:38241 - Hematopoietic progenitor cell (HPC); autologous transplantation
7) CPT:38240 - Hematopoietic progenitor cell (HPC); allogeneic transplantation per donor
8) CPT:44135 - Intestinal allotransplantation; from cadaver donor
9) CPT:47135 - Liver allotransplantation, orthotopic, partial or whole, from cadaver or living donor, any age
10) CPT:48160 - Pancreatectomy, total or subtotal, with autologous transplantation of pancreas or pancreatic islet cells
11) CPT:48554 - Transplantation of pancreatic allograft
12) ICD10CM:Z94.4 - Liver transplant status
13) ICD10CM:Z94.7 - Corneal transplant status
14) ICD10CM:Z94.84 - Stem cells transplant status
15) ICD10CM:Z94.81 - Bone marrow transplant status
16) ICD10CM:Z94.5 - Skin transplant status
17) ICD10CM:Z94.1 - Heart transplant status
18) ICD10CM:Z94.2 - Lung transplant status
19) ICD10CM:Z94.83 - Pancreas transplant status
20) ICD10CM:Z94.6 - Bone transplant status
21) ICD10CM:Z94.82 - Intestine transplant status
22) ICD10CM:Z94.3 - Heart and lungs transplant status
23) ICD10CM:Z94.8 - Other transplanted organ and tissue status
24) ICD10CM:Z94.9 - Transplanted organ and tissue status, unspecified
25) ICD10PCS:0FY - Transplantation
26) ICD10PCS:0BY - Transplantation
27) ICD10PCS:02Y – Transplantation

Adult, Single Renal Transplant with HCV/HIV Exclusion

Exclusion of recipients who were HCV or HIV positive either on or before undergoing the renal transplant procedure using the following codes.

1) ICD10CM:B20 - Human immunodeficiency virus [HIV] disease
2) ICD10CM:B15-B19 - Viral hepatitis

Adult HIV-/HCV- Renal Transplant Recipients with Tacrolimus and Mycophenolate Mofetil/Mycophenolic Only Maintenance Immunosuppression

Recipients must have had tac AND MMF/MPA recorded in their medical record within 1 year on or after any instance of the renal transplant procedure using the following codes:

1) RXNORM:42316 - tacrolimus
2) RXNORM:7145 - mycophenolic acid
3) RXNORM:68149 - mycophenolate mofetil

Recipients were further excluded for any instance of other forms of maintenance immunosuppression within 1 year on or after any instance of the renal transplant procedure using the following codes:

1) RXNORM:1112973 - belatacept
2) RXNORM:1256 - azathioprine
3) RXNORM:3008 - cyclosporine
4) RXNORM:35302 - sirolimus
5) RXNORM:141704 – everolimus

Adult, Early Steroid Withdrawal (ESW) Cohort

After excluding on the previous codes, recipients were selected into the ESW cohort, which was defined as no instance of prednisone administration in the EMR from 7-365 days post-renal transplant procedure using the following codes:

1) RXNORM:8640 – prednisone

Recipients in this cohort must have had greater than or equal to 4 instances of tacrolimus and MMF/MPA codes:

1) RXNORM:42316 - tacrolimus
2) RXNORM:68149 - mycophenolate mofetil
3) RXNORM:7145 - mycophenolic acid

ly, Steroid Continuous Immunosuppression (SCI) Cohort

After excluding on the previous codes, recipients were selected into the SCI cohort, which was defined as prednisone administration in the EMR from 7-365 days post-transplant using the following codes:

1) RXNORM:8640 – prednisone

Recipients in this cohort must have had greater than or equal to 4 instances of tacrolimus and MMF/MPA and prednisone codes:

1) RXNORM:42316 - tacrolimus
2) RXNORM:68149 - mycophenolate mofetil
3) RXNORM:7145 - mycophenolic acid
4) RXNORM:8640 – prednisone

1:1 Propensity Score Matching Criteria

Groups were matched using a 1:1 logistic regression model to proportionally balance cohorts based on the criteria described:

1) AI - Age at Index
2) 2054-5 - Black or African American
3) M - Male
4) N04.1 - Nephrotic syndrome with focal and segmental glomerular lesions
5) N05.1 - Unspecified nephritic syndrome with focal and segmental glomerular lesions
6) N03.3 - Chronic nephritic syndrome with diffuse mesangial proliferative glomerulonephritis
7) M32 - Systemic lupus erythematosus (SLE)
8) E66 - Overweight and obesity
9) E08-E13 - Diabetes mellitus
10) CPT50323: Backbench standard preparation of cadaver donor renal allograft prior to transplantation, including dissection and removal of perinephric fat, diaphragmatic and retroperitoneal attachments, excision of adrenal gland, and preparation of ureter(s), renal vein(s), and renal artery(s), ligating branches, as necessary
11) CPT50325: Backbench standard preparation of living donor renal allograft (open or laparoscopic) prior to transplantation, including dissection and removal of perinephric fat and preparation of ureter(s), renal vein(s), and renal artery(s), ligating branches, as necessary
12) CPT1008104: Backbench reconstruction of cadaver or living donor renal allograft prior to transplantation
13) CPT86900: Blood typing, serologic; ABO
14) CPT86901: Blood typing, serologic; Rh (D)
15) CPT86850: Antibody screen, RBC, each serum technique
16) LOINC80737-0: Calculated panel reactive antibody - Serum
17) LOINC46994-0: HLA-A+B+C (class I) Ab in Serum
18) LOINC882-1: ABO and Rh group [Type] in Blood
19) LOINC24114-1: Epstein Barr virus capsid IgG Ab [Presence] in Serum by Immunoassay
20) LOINC13949-3: Cytomegalovirus IgG Ab [Presence] in Serum or Plasma by Immunoassay
21) LOINC15410-4: Varicella zoster virus IgG Ab [Presence] in Serum by Immunoassay
22) LOINC883-9: ABO group [Type] in Blood
23) LOINC890-4: Blood group antibody screen [Presence] in Serum or Plasma
24) LOINC882-1: ABO and Rh group [Type] in Blood
25) LOINC24114-1: Epstein Barr virus capsid IgG Ab [Presence] in Serum by Immunoassay
26) LOINC13949-3: Cytomegalovirus IgG Ab [Presence] in Serum or Plasma by Immunoassay

27) LOINC15410-4: Varicella zoster virus IgG Ab [Presence] in Serum by Immunoassay
28) LOINC883-9: ABO group [Type] in Blood
29) LOINC890-4: Blood group antibody screen [Presence] in Serum or Plasma

Outcomes Definitions:

1) Renal Transplant Rejection: defined as diagnosed or treatment for rejection.
    a. UMLS:ICD10CM:T86.11, Kidney transplant rejection
    b. NLM:RXNORM:1011, lymphocyte immune globulin, anti-thymocyte globulin
    c. NLM:RXNORM:6902, methylprednisolone (Strength: 100 MG or 125 MG or 500 MG or 1000 MG or 2000 MG)
    d. UMLS:HCPCS:J1459, Injection, immune globulin (privigen), intravenous, non-lyophilized (e.g., liquid), 500 mg
    e. UMLS:HCPCS:J1556, Injection, immune globulin (bivigam), 500 mg
    f. UMLS:HCPCS:J1557, Injection, immune globulin, (gammaplex), intravenous, non-lyophilized (e.g., liquid), 500 mg
    g. UMLS:HCPCS:J1561, Injection, immune globulin, (gamunex-c/gammaked), non-lyophilized (e.g., liquid), 500 mg
    h. UMLS:HCPCS:J1566, Injection, immune globulin, intravenous, lyophilized (e.g., powder), not otherwise specified, 500 mg
    i. UMLS:HCPCS:J1568, Injection, immune globulin, (octagam), intravenous, non-lyophilized (e.g., liquid), 500 mg
    j. UMLS:HCPCS:J1569, Injection, immune globulin, (gammagard liquid), non-lyophilized, (e.g., liquid), 500 mg
    k. UMLS:HCPCS:J1572, Injection, immune globulin, (flebogamma/flebogamma dif), intravenous, non-lyophilized (e.g., liquid), 500 mg
    l. UMLS:HCPCS:J1599, Injection, immune globulin, intravenous, non-lyophilized (e.g., liquid), not otherwise specified, 500 mg

2) Graft Failure: defined as any instance of an laboratory measured eGFR ≤ 15 mL/min/{1.73_m2}
    a. UMLS:LNC:62238-1, Glomerular filtration rate/1.73 sq M.predicted [Volume Rate/Area] in Serum, Plasma or Blood by Creatinine-based formula (CKD-EPI) (at most 15.00 mL/min/{1.73_m2} (most recent occurrence))
3) Mortality: defined using TriNetX demographic data of deceased
    a. Deceased
4) CMV Viremia: defined as diagnosed cytomegaloviral disease or laboratory confirmed CMV viral load (at least 1000.00 {copies}/mL or at least 3.00 {Log_IU}/mL)
    a. UMLS:LNC:30247-1, Cytomegalovirus DNA [#/volume] (viral load) in Serum or Plasma by NAA with probe detection (at least 1000.00 {copies}/mL (most recent occurrence))
    b. UMLS:LNC:29604-6, Cytomegalovirus DNA [#/volume] (viral load) in Blood by NAA with probe detection (at least 1000.00 {copies}/mL (most recent occurrence))
    c. UMLS:LNC:72493-0, Cytomegalovirus DNA [Units/volume] (viral load) in Plasma by NAA with probe detection (at least 1000.00 [IU]/mL (most recent occurrence))

    d. UMLS:LNC:33006-8, Cytomegalovirus DNA [#/volume] (viral load) in Specimen by NAA with probe detection (at least 1000.00 {copies}/mL (most recent occurrence))
    e. UMLS:LNC:34720-3, Cytomegalovirus DNA [Units/volume] (viral load) in Specimen by NAA with probe detection (at least 1000.00 [IU]/mL (most recent occurrence))
    f. UMLS:LNC:24041-6, Cytomegalovirus DNA [Units/volume] (viral load) in Specimen by Probe with signal amplification (at least 1000.00 [arb'U]/mL (most recent occurrence))
    g. UMLS:LNC:49347-8, Cytomegalovirus DNA [#/volume] (viral load) in Urine by NAA with probe detection (at least 1000.00 {copies}/mL (most recent occurrence))
    h. UMLS:LNC:49351-0, Cytomegalovirus DNA [#/volume] (viral load) in Tissue by NAA with probe detection (at least 1000.00 {copies}/mL (most recent occurrence))
    i. UMLS:LNC:54206-8, Cytomegalovirus DNA [Log #/volume] (viral load) in Serum or Plasma by NAA with probe detection (at least 3.00 {Log_copies}/mL (most recent occurrence))
    j. UMLS:LNC:53763-9, Cytomegalovirus DNA [Log #/volume] (viral load) in Specimen by NAA with probe detection (at least 3.00 {Log_copies}/mL (most recent occurrence))
    k. UMLS:LNC:72494-8, Cytomegalovirus DNA [log units/volume] (viral load) in Plasma by NAA with probe detection (at least 3.00 {Log_IU}/mL (most recent occurrence))
    l. UMLS:LNC:96396-7, Cytomegalovirus DNA [log units/volume] (viral load) in Specimen by NAA with probe detection (at least 3.00 {Log_IU}/mL (most recent occurrence))
    m. UMLS:ICD10CM:B25, Cytomegaloviral disease

5) EBV viremia: defined as diagnosed infectious mononucleosis or laboratory confirmed EBV viral load (at least 1000.00 {copies}/mL or at least 3.00 {Log_IU}/mL)
    a. UMLS:LNC:32585-2, Epstein Barr virus DNA [#/volume] (viral load) in Specimen by NAA with probe detection (at least 1000.00 {copies}/mL (most recent occurrence))
    b. UMLS:LNC:53774-6, Epstein Barr virus DNA [Log #/volume] (viral load) in Specimen by NAA with probe detection (at least 3.00 {Log_copies}/mL (most recent occurrence))
    c. UMLS:LNC:36923-1, Epstein Barr virus DNA [#/volume] (viral load) in Blood by NAA with probe detection (at least 1000.00 {copies}/mL (most recent occurrence))
    d. UMLS:LNC:93840-7, Epstein Barr virus DNA [Units/volume] (viral load) in Blood by NAA with probe detection (at least 1000.00 [IU]/mL (most recent occurrence))
    e. UMLS:LNC:93841-5, Epstein Barr virus DNA [log units/volume] (viral load) in Blood by NAA with probe detection (at least 3.00 {Log_IU}/mL (most recent occurrence))
    f. UMLS:LNC:43730-1, Epstein Barr virus DNA [Units/volume] (viral load) in Serum or Plasma by NAA with probe detection (at least 1000.00 [IU]/mL (most recent occurrence))
    g. UMLS:LNC:47982-4, Epstein Barr virus DNA [#/volume] (viral load) in Serum or Plasma by NAA with probe detection (at least 1000.00 {copies}/mL (most recent occurrence))
    h. UMLS:ICD10CM:B27.9, Infectious mononucleosis, unspecified

6) BK viremia: defined as laboratory confirmed BK viral load (at least 1000.00 {copies}/mL or at least 3.00 {Log_IU}/mL)
    a. UMLS:LNC:41479-7, BK virus DNA [#/volume] (viral load) in Serum or Plasma by NAA with probe detection (at least 1000.00 {copies}/uL (most recent occurrence))

b. UMLS:LNC:41480-5, BK virus DNA [#/volume] (viral load) in Urine by NAA with probe detection (at least 1000.00 {copies}/uL (most recent occurrence))
c. UMLS:LNC:48309-9, BK virus DNA [#/volume] (viral load) in Specimen by NAA with probe detection (at least 1000.00 {copies}/mL (most recent occurrence))
d. UMLS:LNC:32284-2, BK virus DNA [Units/volume] (viral load) in Serum or Plasma by NAA with probe detection (at least 1000.00 [IU]/mL (most recent occurrence))
e. UMLS:LNC:49345-2, BK virus DNA [#/volume] (viral load) in Blood by NAA with probe detection (at least 1000.00 {copies}/mL (most recent occurrence))
f. UMLS:LNC:32285-9, BK virus DNA [Units/volume] (viral load) in Urine by NAA with probe detection (at least 1000.00 [IU]/mL (most recent occurrence))
g. UMLS:LNC:49344-5, BK virus DNA [#/volume] (viral load) in Cerebral spinal fluid by NAA with probe detection (at least 1000.00 {copies}/mL (most recent occurrence))
h. UMLS:LNC:42587-6, BK virus DNA [Units/volume] (viral load) in Specimen by NAA with probe detection (at least 1000.00 [arb'U]/mL (most recent occurrence))
i. UMLS:LNC:43201-3, BK virus DNA [Log #/volume] (viral load) in Specimen by NAA with probe detection (at least 3.00 {Log_copies}/mL (most recent occurrence))
j. UMLS:LNC:90924-2, BK virus DNA [Log #/volume] (viral load) in Urine by NAA with probe detection (at least 3.00 {Log_copies}/mL (most recent occurrence))
k. UMLS:LNC:44805-0, BK virus DNA [Log #/volume] (viral load) in Serum or Plasma by NAA with probe detection (at least 3.00 {Log_copies}/mL (most recent occurrence))

7) JC viremia: defined as laboratory confirmed JC viral load (at least 1000.00 {copies}/mL or at least 3.00 {Log_IU}/mL)
   a. UMLS:LNC:49412-0, JC virus DNA [#/volume] (viral load) in Specimen by NAA with probe detection (at least 1000.00 {copies}/mL (most recent occurrence))
   b. UMLS:LNC:49414-6, JC virus DNA [#/volume] (viral load) in Blood by NAA with probe detection (at least 1000.00 {copies}/mL (most recent occurrence))
   c. UMLS:LNC:49411-2, JC virus DNA [#/volume] (viral load) in Urine by NAA with probe detection (at least 1000.00 {copies}/mL (most recent occurrence))
   d. UMLS:LNC:49545-7, JC virus DNA [Units/volume] (viral load) in Cerebral spinal fluid by NAA with probe detection (at least 1000.00 [arb'U]/mL (most recent occurrence))
   e. UMLS:LNC:49410-4, JC virus DNA [#/volume] (viral load) in Cerebral spinal fluid by NAA with probe detection (at least 1000.00 {copies}/mL (most recent occurrence))
   f. UMLS:LNC:49413-8, JC virus DNA [#/volume] (viral load) in Serum or Plasma by NAA with probe detection (at least 1000.00 {copies}/mL (most recent occurrence))
   g. UMLS:LNC:100685-7, JC virus DNA [Log #/volume] (viral load) in Serum or Plasma by NAA with probe detection (at least 3.00 {Log_copies}/mL (most recent occurrence))

8) VZV Viremia: defined as diagnosed varicella (herpes zoster) infection or laboratory confirmed VZV viral load (at least 1000.00 {copies}/mL)
   a. ICD10CM:B02 - Zoster [herpes zoster]
   b. LNC:49451-8 - Varicella zoster virus DNA [#/volume] (viral load) in Specimen by NAA with probe detection (at least 1000.00 {copies}/mL (most recent occurrence))

    c. LNC:49455-9 - Varicella zoster virus DNA [#/volume] (viral load) in Bronchoalveolar lavage by NAA with probe detection (at least 1000.00 {copies}/mL (most recent occurrence))
    d. LNC:47003-9 - Varicella zoster virus DNA [#/volume] (viral load) in Serum or Plasma by NAA with probe detection (at least 1000.00 {copies}/mL (most recent occurrence))
    e. LNC:47002-1 - Varicella zoster virus DNA [#/volume] (viral load) in Cerebral spinal fluid by NAA with probe detection (at least 1000.00 {copies}/mL (most recent occurrence))

9) Composite viremia: composite of diagnosed or laboratory confirmed viral load (load (at least 1000.00 {copies}/mL or at least 3.00 {Log_IU}/mL)
    a. All previously described CMV viremia codes
    b. All previously described EBV viremia codes
    c. All previously described BK viremia codes
    d. All previously described JC viremia codes
    e. All previously described VZV viremia codes

10) Tacrolimus Trough: measured tacrolimus blood levels at 1 month, 3 months, 6 months, 12 months
    a. 11253-2: Tacrolimus [Mass/volume] in Blood ((most recent occurrence))

11) eGFR: measured eGFR CKD-EPI values at 1 month, 3 months, 6 months, and 12 months
    a. 62238-1: Glomerular filtration rate/1.73 sq M.predicted [Volume Rate/Area] in Serum, Plasma or Blood by Creatinine-based formula (CKD-EPI) ((most recent occurrence))

12) MMF: measured mycophenolate levels at 1, 3, 6, and 12 months post-transplant
    a. UMLS:LNC:23905-3, Mycophenolate [Mass/volume] in Serum or Plasma ((most recent occurrence))

13) DGF: need for hemodialysis in the first week post-transplant
    a. CPT® 90935, Under Hemodialysis Procedures

14) Sepsis: defined as diagnosed sepsis
    a. UMLS:ICD10CM:A41.9, Sepsis, unspecified organism

15) Pyelonephritis/KTx infection: defined as diagnosed peylonephritis or kidney transplant infection
    a. UMLS:ICD10CM:N10: acute pyelonephritis
    b. UMLS:ICD10CM:T86.13, Kidney transplant infection

16) Post-transplant Diabetes Mellitus: defined as hemoglobin A1c >6.5%
    a. TNX Curated: Hemoglobin A1c/hemoglobin.total in blood (most recent value > 6.5%)
